# Two New Mylagaulid Rodents from the Early Miocene of China

**DOI:** 10.1371/journal.pone.0159445

**Published:** 2016-08-03

**Authors:** Xiaoyu Lu, Xijun Ni, Lüzhou Li, Qiang Li

**Affiliations:** 1 Key Laboratory of Vertebrate Evolution and Human Origins, Institute of Vertebrate Paleontology and Paleoanthropology, Chinese Academy of Sciences, 142 Xi Zhi Men Wai Street, Beijing, 100044, China; 2 CAS Center for Excellence in Tibetan Plateau Earth Sciences, Beijing, 100101, China; New York Institute of Technology College of Osteopathic Medicine, UNITED STATES

## Abstract

Mylagaulid fossorial rodents are a common component of North American Miocene fossil faunas. However outside of North America, only three species are known from Asia. Here we report two new mylagaulids, *Irtyshogaulus minor* gen. et sp. nov. and *Irtyshogaulus major* gen. et sp. nov., recovered from early Miocene sediments in the Junggar Basin in northwestern China. The two new taxa are small-sized, high-crowned promylagauline rodents. Their lower molars possess high metastylid crests, small mesostylids, broad and posterolingually expanded labial inflections, and transversely extending metalophid IIs. The mesoconid is absent in both species. The anterior and posterior fossettids are large and equally developed. Their upper M1-2s possess a square occlusal surface with five deep fossettes. The two new taxa are distinguished from each other mainly by their size, the morphology of fossettes and fossettids, development of mesial and distal lophs, posterior reduction of M3, and the orientation of m2 hypolophid. Our phylogenetic analysis indicates that *Irtyshogaulus* and *Lamugaulus* (another early Miocene Asian mylagaulid) are sister taxa. The two genera are nested among the North American promylagaulines, and share a common ancestor from North America, indicating early Miocene intercontinental dispersal within this clade of rodents.

## Introduction

Mylagaulidae is a group of extinct fossorial rodents that were common in open environments across the Miocene of western North America. Derived forms have broad and robust skulls, long digging claws, short forelimbs, heavily built limb girdles, and broad limb joint articulations. All of these features are typical adaptations to a fossorial life habit [1,2]. Some genera of Mylagaulidae, such as *Umbogaulus*, *Ceratogaulus*, and *Epigaulus*, are well-known for their very peculiar, paired horns developed on the nasals and premaxillae [3–5]. Among rodents, these horns are unique, and the hypothesized function(s) of the horns has attracted wide interest and speculation among paleontologists and evolutionary biologists [1]. Mylagaulids also are characterized by their very specialized dentition. In many mylagaulids, the upper and lower last premolars become progressively enlarged and hypsodont. In some species, the P4/p4 are so enlarged that the first molar, and even the second molar, are pushed out during the eruption of the P4/p4, leaving only one or two molars functional [1,6,7].

Mylagaulids originated in North America [6–8] during the Oligocene (Arikareean-1 North American Land Mammal Age), by about 30 Ma [6–8]. Through the Miocene, mylagaulids diversified into the 13 genera and approximately 30 species known [7]. However, only two genera (*Mylagaulus* and *Epigaulus*) survived into the beginning of the Pliocene, before extinction of the clade.

Outside of North America, mylagaulids are extremely rare, and only three species are known, represented by a small number of isolated teeth from middle to high latitude areas in northern Asia. They are *Tschalimys ckhikvadzei* Shevyreva, 1971, *Simpligaulus yangi* Wu et al., 2013, and *Lamugaulus olkhonensis* Tesakov and Lopatin, 2015. *Tschalimys ckhikvadzei* is known from the early middle Miocene Sarybulak Formation in the Zaisan Basin of Kazakhstan and the Halamagai Formaion at the Tieersihabahe locality in northern Junggar Basin in China [9,10]. *Simpligaulus*, is known only from an isolated p4, from the Halamagai Formation at the Tieersihabahe locality [10]. The third species, *Lamugaulus olkonensis* is from the early Miocene Khalagay Formation in the Irkutsk region of Russia [11].

Adding to that Asian record are two new mylagaulid species (described below) discovered in early Miocene sediments at the XJ2006 locality approximately 35 km northwest of Burqin Town in the northwestern Junggar Basin, Xinjiang Province, China. The fossils were collected from a lens of pebbly coarse sandstone in the basal layer of an unnamed rock unit consisting of grayish to blackish yellow fluvial sandstone and sandy mudstone. These fluvial sediments overlie the brightly–colored Oligocene Irtysh River Formation. A diverse small mammal community is known from the locality [12,13], and a preliminary study identified two lagomorphs, three glirids (*Miodyromys asiamediae* Maridet et al., 2011, *Microdyromys* aff. *orientalis* Wu, 1986, and one unidentified species of *Eliomys*), four eomyids (*Asianeomys* aff. *engesseri* Wu, 1986, *Asianeomys* sp., *Keramidomys* sp., and an unidentified eomyid), three cricetids (*Democricetodon* sp., *Cricetodon* sp., and microtoid cricetid *Primoprismus fejfari*), and a new species of *Ansomys* [13]. Correlation of the mammalian fauna with other better known Chinese faunas points to an age within the middle Shanwangian Asian Land Mammal Age (late early Miocene), roughly 17–18 Ma [12,13].

## Material and Methods

### Material

The mylagaulid specimens reported here were obtained by screen-washing the fossiliferous sediments. All specimens are publicly deposited and accessible in the collections of the Institute of Vertebrate Paleontology and Paleoanthropology (IVPP), Chinese Academy of Sciences (142 Xi Zhi Men Wai Street, Beijing, China). The specimen numbers include, IVPP V 20328, V 20329.1–15, V 20809, V 20810.1–8. All of the specimens were collected during the field expeditions supported by the Chinese Academy of Sciences, the Ministry of Science and Technology of China, and the National Natural Science Foundation of China. No permits were required for this study, which complied with all relevant regulations.

### Methods

**Fig 1:**
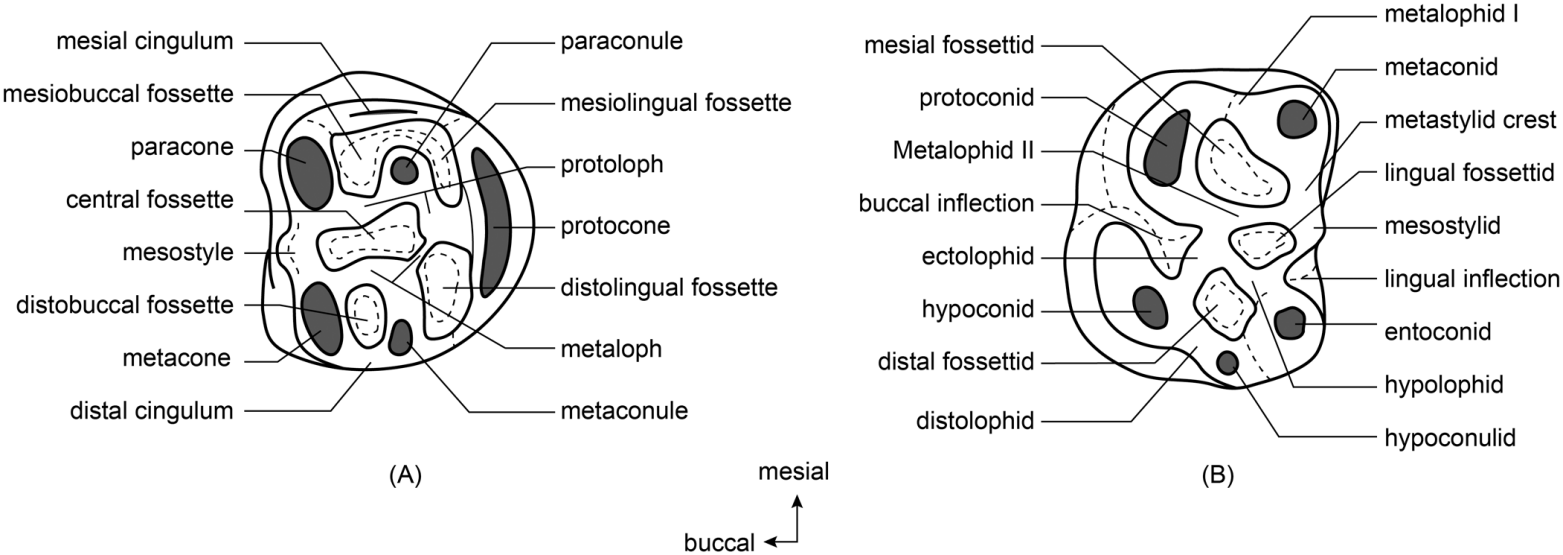
Terminology used for the mylagaulid molars. (A), Upper molar; (B), Lower molar.

Dental terminology (Fig 1) is modified from that of Rensberger [14] and Wu et al. [10].

Specimens were measured under the ZEN Pro 2012 system with a Zeiss stereo-microscope (Discovery V20), and were calibrated from digital calipers.

The specimen images were produced using the 225 kV micro-CT facility at the Key Laboratory of Vertebrate Evolution and Human Origins of the Chinese Academy of Sciences. All the specimens were CT scanned with beam energy of 140 kV and a flux of 100 uA at a detector resolution of 42.3 um per pixel using a 360° rotation with a step size of 0.5° and an unfiltered aluminium reflection target. Three-dimensional reconstructions were produced in the VGStudio Max software 2.2.

The new mylagaulid specimens represent two new species placed into one new genus. The two species were scored for an updated phylogenetic analysis based on the data matrix developed by Hopkins [8] for the most comprehensive investigation on the phylogeny of the superfamily Aplodontoidea (S1 Text). In addition to the new mylagaulid genus, we scored all previously known Asian mylagaulids including *Tschalimys*, *Simpligaulus*, and *Lamugaulus*. In addition, we added *Brachygaulus* to the analysis because Korth and Tabrum [15] suggested that the genus may represent the ancestral mylagaulid morphotype. In total, 250 characters were scored for 111 taxa (S1 Text).

We followed the strategy of Hopkins [8] by selecting *Ischyromys typus*, *Paramys delicatus*, and *Reithroparamys delicatissimus* as outgroup taxa, and by using the semi-ordered character configuration. The data matrix was edited in Mesquite v3.03 software [16] and saved in the NEXUS format. Parsimony analysis was undertaken using TNT (Tree analysis using New Technology), a parsimony analysis program subsidized by the Willi Hennig Society [17]. We ran multiple replications, using sectorial searches, drifting, ratchet and fusing combined, a searching strategy previously used by Ni et al. [18]. Random sectorial search, constraint sectorial search and exclusive sectorial search were set. Ten cycles of tree drift, 10 cycles of ratchet, and 10 cycles of tree fusing were performed in the search. Default parameter settings were used for random sectorial search, constraint sectorial search, exclusive sectorial search, tree drifting, ratchet, and fusing. The search level was set as 10 for 112 taxa. Optimal scores were searched with 10,000 replications. Some characters were set as ordered (as did Hopkins [8]). All characters had equal weight. One positive constraint was used to keep the ingroup as a monophyletic unit.

About one and a half hours were required to finish the search, with more than 188 billion rearrangements examined. Fifty eight trees with a best score of 1585 were retained. The best trees and the Majority-rule consensus of these trees were described in PAUP* [19].

### Institutional Abbreviation

IVPP, Institute of Vertebrate Paleontology and Paleoanthropology, Chinese Academy of Sciences (Beijing, China).

### Nomenclatural Acts

The electronic edition of this article conforms to the requirements of the amended International Code of Zoological Nomenclature, and hence the new names contained herein are available under that Code from the electronic edition of this article. This published work and the nomenclatural acts it contains have been registered in ZooBank, the online registration system for the ICZN. The ZooBank LSIDs (Life Science Identifiers) can be resolved and the associated information viewed through any standard web browser by appending the LSID to the prefix “http://zoobank.org/”. The LSID for this publication is: urn:lsid:zoobank.org:pub:362894B9-30B4-4C74-8A29-CE6B4164C1C0. The electronic edition of this work was published in a journal with an ISSN, and has been archived and is available from the following digital repositories: PubMed Central, LOCKSS.

## Results

### Systematic paleontology

Rodentia Bowdich, 1821

Aplodontoidea Brandt, 1855

Mylagaulidae Cope, 1881

Promylagaulinae Rensberger, 1980

#### Included Genera

*Promylagaulus* McGrew, 1941; *Trilaccogaulus* Korth 1992; *Lamugaulus* Tesakova and Lopatin, 2015; *Irtyshogaulus* gen. nov.

#### Emended Diagnosis

Cheek teeth mesodont, prismatic, and single-rooted. P3 present, buccolingually compressed. P4 with buccolingually expanded anterocone; P4 distolingual fossette mesiodistally aligned. P4-M3 protocone mesiodistally elongated and buccolingually flattened; paracone and metacone with high and thick mesial and distal crests; paracone and metacone buccal side convex; cingula wall-like; paraconule and metaconule massive and pillar-like; mesostyle small; presenting 4–5 major fossettes in early wear; at least one fossette (distolingual fossette) remained in deep wear stage; and mesial dentine tract present. M3 smaller than M1-2. p4-m3 protoconid crescent, metalophid I and metalophid II present; metaconid buccolingually narrow, metastylid small; high and wall-like crest connecting metaconid and metastylid; hypoconid mesiobuccally expanded; distolophid buccally merged into hypoconid and lingually jointing entoconid; ectolophid high and thick; mesoconid absent or very small; present 3–4 fossettids; and at least one fossettid remained in deep wear stage. Buccal inflection becomes shallow with wear.

### *Irtyshogaulus* gen. nov.

urn:lsid:zoobank.org:act:58EF94AF-25B4-423C-9017-8B9639759AD0

#### Type Species

*Irtyshogaulus minor* sp. nov.

#### Diagnosis

Small sized, mesodont promylagauline rodent. Lower molars present high metastylid crest, small mesostylid, broad and posterolingually expanded labial inflection, and a transversely extending metalophid II. Mesoconid absent. Anterior and posterior fossettids large and equally developed. Upper M1-2 present square occlusal surface with five deep fossettes. Two fossettes remain in very deep wear stage.

#### Etymology

The generic name derives from the Irtysh River where the specimens were found and from ‘gaulus’ (Greek for bowl), the generic root for taxa in the Family Mylagaulidae.

### *Irthyshogaulus minor* sp. nov.

**Fig 2:**
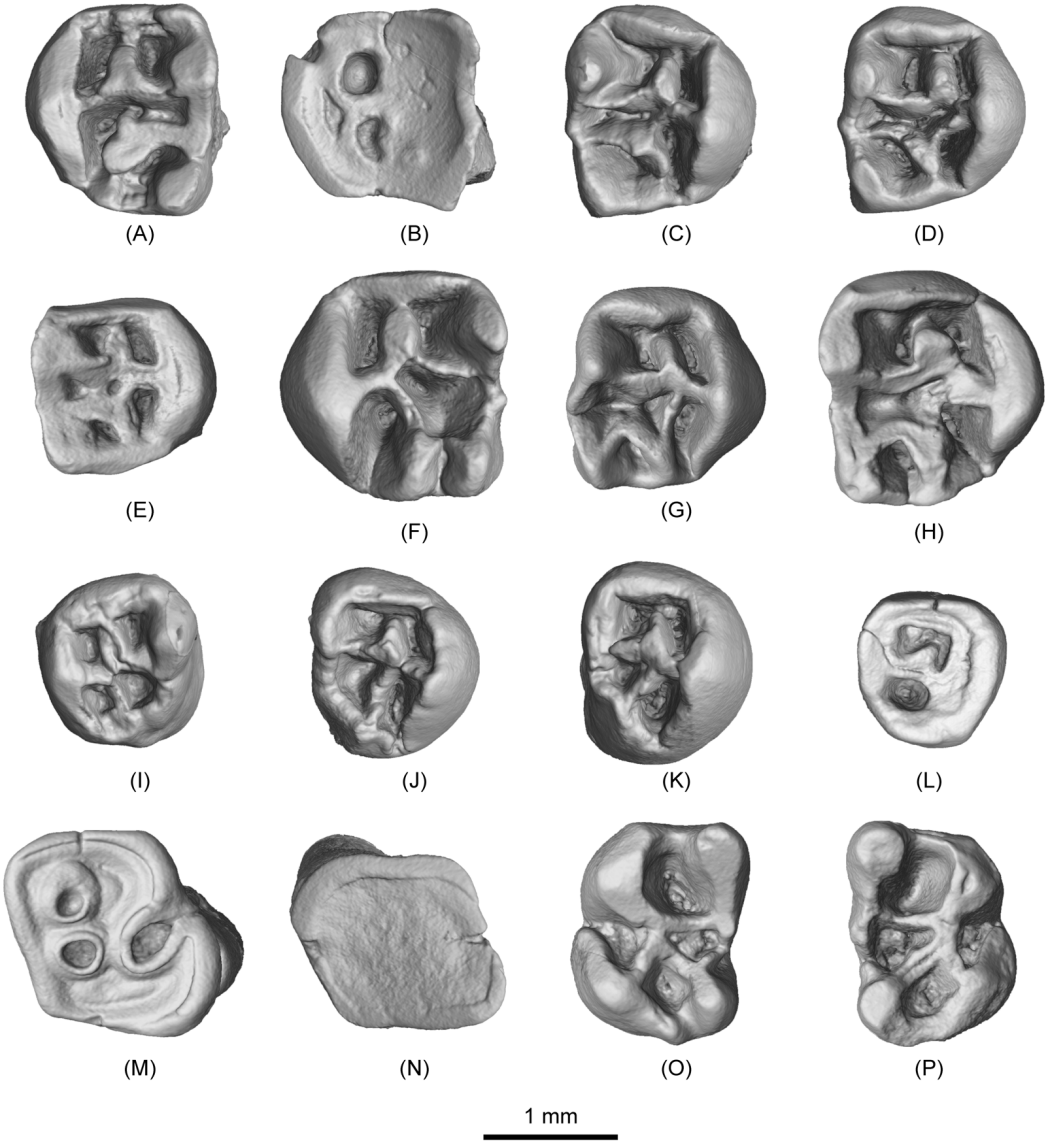
Upper and lower teeth of *Irtyshogaulus minor* gen. et sp. nov. (A), V 20328, left M1 (holotype); (B), V 20329.1, left M1; (C)-(E), V 20329.2–4, right M1s; (F), V 20329.5, left M2; (G)-(H), V 20329.6–7, right M2s; (I), V 20329.8, left M3; (J)-(L), V 20329.9–11, right M3s; (M)-(N), V 20329.12–13, right m1s; (O), V 20329.14, left m2; (P), V 20329.15, right m2; Scale bar, 1 mm.

urn:lsid:zoobank.org:act:7B52DA62-8CF8-451D-9239-997EB27F3379

(Fig 2; Table 1; S1–S6 Movies)

**Table 1:** Measurements for *Irtyshogaulus minor* gen. et sp. nov.

Specimen number	Tooth	Length (mm)	Width (mm)
IVPP V 20328	left M1	1.57	1.33
IVPP V 20329.1	left M1	1.50	1.58
IVPP V 20329.2	right M1	1.57	1.48
IVPP V 20329.3	right M1	1.53	1.60
IVPP V 20329.4	right M1	1.31	1.39
IVPP V 20329.5	left M2	1.71	1.67
IVPP V 20329.6	right M2	1.50	1.59
IVPP V 20329.7	right M2	1.74	1.81
IVPP V 20329.8	left M3	1.29	1.23
IVPP V 20329.9	right M3	1.41	1.23
IVPP V 20329.10	right M3	1.57	1.48
IVPP V 20329.11	right M3	1.16	1.17
IVPP V 20329.12	right m1	1.53	1.30
IVPP V 20329.13	right m1	1.45	1.46
IVPP V 20329.14	left m2	1.65	1.05
IVPP V 20329.15	right m2	1.67	1.23

#### Type Specimen

IVPP V 20328, a left M1.

#### Referred Specimens

IVPP specimens: V 20329.1, a left M1; V 20329.2–4, 3 right M1s; V 20329.5, a left M2; V 20329.6–7, 2 right M2s; V 20329.8, a left M3; V 20329.9–11, 3 right M3s; V 20329.12–13, 2 right m1s; V 20329.14, a left m2; and V 20329.15, a right m2.

#### Locality and age

Locality XJ200604, northwestern Junggar Basin, Xinjiang, China. Early Miocene.

#### Diagnosis

Small species of *Irthyshogaulus*. Molars present relatively bigger fossettes and fossettids. M1-2 with thick and high mesial loph, but relatively weak distal loph. M3 distally reduced. Metaconule absent or very small. Lower m2 hypolophid extending mesiobuccally. Lingual fossettid is triangular in shape.

#### Etymology

The specific epithet refers to the relatively smaller size of this species.

#### Description

The M1s attributed to *Irtyshogaulus minor* represent different wear stages. The occlusal facet is roughly square, with similar width and length. As in *Promylagaulus*, *Trilaccogaulus*, and *Lamugaulus*, the mesial cingulum is thick and high, forming a high mesial edge of the tooth, but the distal cingulum in *I*. *minor* is low, differing from those taxa. The large and buccolingually compressed protocone connects with the mesial and distal cingula with its mesial and distal crests respectively. Compared to *Lamugaulus*, the protocone of *I*. *minor* is more mesiodistally expanded. The wear facets of the two protocone crests are equally developed, and proportionally longer than those in *Lamugaulus*. The paracone and metacone are conical. Their mesial and distal crests are connected into a complete wall defining the buccal tooth border. Such a complete and straight buccal wall also is present in *Promylagaulus* and *Trilaccogaulus*. *Lamugaulus* has more conical paraconid and metaconid with relatively lower mesial and distal crests. The paraconule and metaconule are two pillar-like cusps located in the middle of the tooth crown. This feature is present in all primitive mylagaulids. The mesial side of the base of the paraconule is fused with the mesial cingulum. The metaconule extends distally and is fused with the distal cingulum at the base. In early wear, the connections between paraconule, metaconule, and the cingula are low and thin. Large conules tend to fuse with the high and thick cingula in promylagaulines, but in *Lamugaulus*, the distal side of the metaconule is free and does not connect the distal cingulum. The protoloph is a thick and high ridge developed from the buccal side of the protocone that connects the distal part of the paraconule with the protocone lingually and the paracone buccally. The metaloph is not a straight ridge. Its lingual part arises from the protoloph close to the protocone. It turns buccodistally to join the metaconule. The buccal part of the metaloph is high and straight. It connects the mesial part of the metaconule to the buccal tooth margin. In all promylagaulines, the lingual end of the metaloph joins the protoloph instead of the protocone. Along the buccal side and between the paracone and metacone, a very small mesostyle is present. Having a small mesostyle is a feature that distinguishes promylagaulines from aplodontids, where instead the mesostyle forms a sharp ridge along the buccal tooth side. The M1s bear five fossettes (the mesiolingual, mesiobuccal, distolingual, distobuccal, and central fossettes). The mesiolingual and mesiobuccal fossettes are roughly square shaped. The distolingual and distobuccal fossettes are subtriangular in shape. The buccal fossettes are slightly larger than their lingual counter fossettes. The central fossette is mesiodistally narrow, but transversely wide. When the tooth is deeply worn, the central fossette is separated into two small fossettes by the confluence of the protoloph and metaloph. In a very deeply worn tooth (IVPP V 20329.1), the mesiolingual and the distolingual fossettes are present. In deeply worn upper molars of *Promylagaulus* and *Trilaccogaulus*, only one fossette is present, and that fossette probably is the distolingual fossette.

Some teeth are identified as M2s because the distal parts of the M2 is somewhat reduced and simplified. Roughly the M2s of *I*. *minor* are about the same size as the M1s. Different from the M1, the M2 has relatively weak distal cingula, which partly enclose the distal fossettes. The paraconule has a weaker connection with the mesial cingulum. Therefore, the mesiolingual and mesiobuccal fossettes tend to connect to each other. The metaconule is more distally positioned and nearly completely fused with the distal cingulum. The lingual part of the metaloph makes a distal U-turn to join the distally positioned metaconule. The buccal part of the metaloph also is curved and connects to the metacone instead of the mesial crest of the metacone. The central fossette is significantly enlarged, and the distobuccal fossette is significantly reduced.

The M3 is much smaller than both the M1 and M2. The occlusal surface of the tooth is oval in shape, with a rounded lingual side and a relatively straight buccal side. The protocone, paracone, and metacone are all reduced to crest-like structures. The paraconule, metaconule, protoloph, and metaloph are not differentiated. There are only two ridges that form a cross and separate four fossettes on the occlusal surface.

Two deeply worn lower molars (IVPP V 20329.12–13) are identified as m1s, and the single root in those two teeth is mesiodistally more compressed than in the teeth identified as m2s. The two teeth all have trapezoidal occlusal surfaces, with their distal sides slightly wider than their mesial sides. One tooth was worn to the base of the crown with no crest or fossette remaining. Another younger individual shows a deep and distally expanded buccal inflection, a feature similar to *Lamugaulus*. Compared with *Promylagaulus* and *Trilaccogaulus*, the buccal inflection in the m1 of *I*. *minor* is distally more expanded, because the m1s of *Promylagaulus* and *Trilaccogaulus* bear a strong ectomesolophid, extending from the ectolophid and buccally dividing the buccal inflection. Two round fossae correspond to the mesial fossettid and lingual fossettid.

Two teeth (IVPP V 20329.14–15) are identified as m2s. Proportionally they are longer than the m1s. The m2 has four major cusps (the protoconid, metaconid, hypoconid, and entoconid). The protoconid is crescent shaped. Its mesial arm extends lingually to join the metaconid, and forms a high and thick metalophid I. Its distal arm extends parallel to metalophid I to connect the distal crest of the metaconid, and therefore it becomes the metalophid II. In *Promylagaulus* and *Trilaccogaulus*, the metalophid II has a mesial spur that joins the metaconid, and in *I*. *minor* and *Lamugaulus*, the metalophid II is a straight crest without any divisions. The metaconid is higher and sharper than the protoconid. Its mesial side is round and smooth. Its distal side tapers into a high ridge, equivalent to the metastylid crest. At the distal end of the metastylid crest, there is a small mesostylid. The shape of the metaconid and the strong connection between the metaconid and metastylid in *I*. *minor* is similar to those features in *Lamugaulus*. In *Promylagaulus* and *Trilaccogaulus*, the metaconid is less conical and more crest-like. The hypoconid is about the same shape and size as the protoconid. Its mesiobuccal side extends as a short and blunt ridge. Its distolingual side bears a long arm to form the distolophid. Compared to *Promylagaulus* and *Trilaccogaulus*, the mesiobuccal expansion in *I*. *minor* is greater and similar to that in *Lamugaulus*. Mesiolingually, the oblique ectolophid originates from the hypoconid and extends to the join the distolingual side of the protoconid. In *Promylagaulus* and *Trilaccogaulus*, the ectolophid is straight and slightly more buccally positioned. In *Lamugaulus*, the ectolophid is as strong as in *I*. *minor*, but the orientation of this crest in *Lamugaulus* is mesiobuccal-distolingual. The shape of the entoconid is similar to that of other promylagaulines. Its mesiobuccal side tapers into a high hypolophid, which extends obliquely to connect to the mesial part of the ectolophid. From its mesiolingual side, a small and short ridge is present that joins the metastylid crest. Between the protoconid and metaconid, a deep and round mesial fossettid is enclosed by the metalophid I and II. This morphology forms a deep and round mesial fossettid that also is present in *Lamugaulus*. In *Promylagaulus* and *Trilaccogaulus*, the mesial fossettid is divided by the mesial spur of the metalophid II. Between the hypoconid and entoconid, a similar deep but smaller fossa (the distal fossettid), is enclosed by the ectolophid, hypolophid, and distolophid. In *Lamugaulus*, the distal fossettid is about the same size as the mesial fossettid, and in *Promylagaulus* and *Trilaccogaulus*, the distal fossettid is saliently larger than the divided mesial fossettid. Between the protoconid and hypoconid, the buccal inflection is deep and distally expanded, forming a semi-closed fossa. As in *Lamugaulus*, this fossa is deep and round. The buccal inflection is shallower in *Promylagaulus* and *Trilaccogaulus*, partly because the ectolophid in these two taxa is more buccally positioned and partly because the developed ectomesolophid usually joins the mesiobuccal expansion of the hypoconid and divides the buccal inflection. Opposite the buccal inflection, the lingual fossettid is present as a triangular fossa enclosed by the metalophid II, and the hypolophid and metastylid crests. A shallow lingual inflection is present mesiolingual to the entoconid. Compared to other promylagaulines, the lingual inflection in *I*. *minor* is much shallower.

### *Irtyshogaulus major* sp. nov.

**Fig 3:**
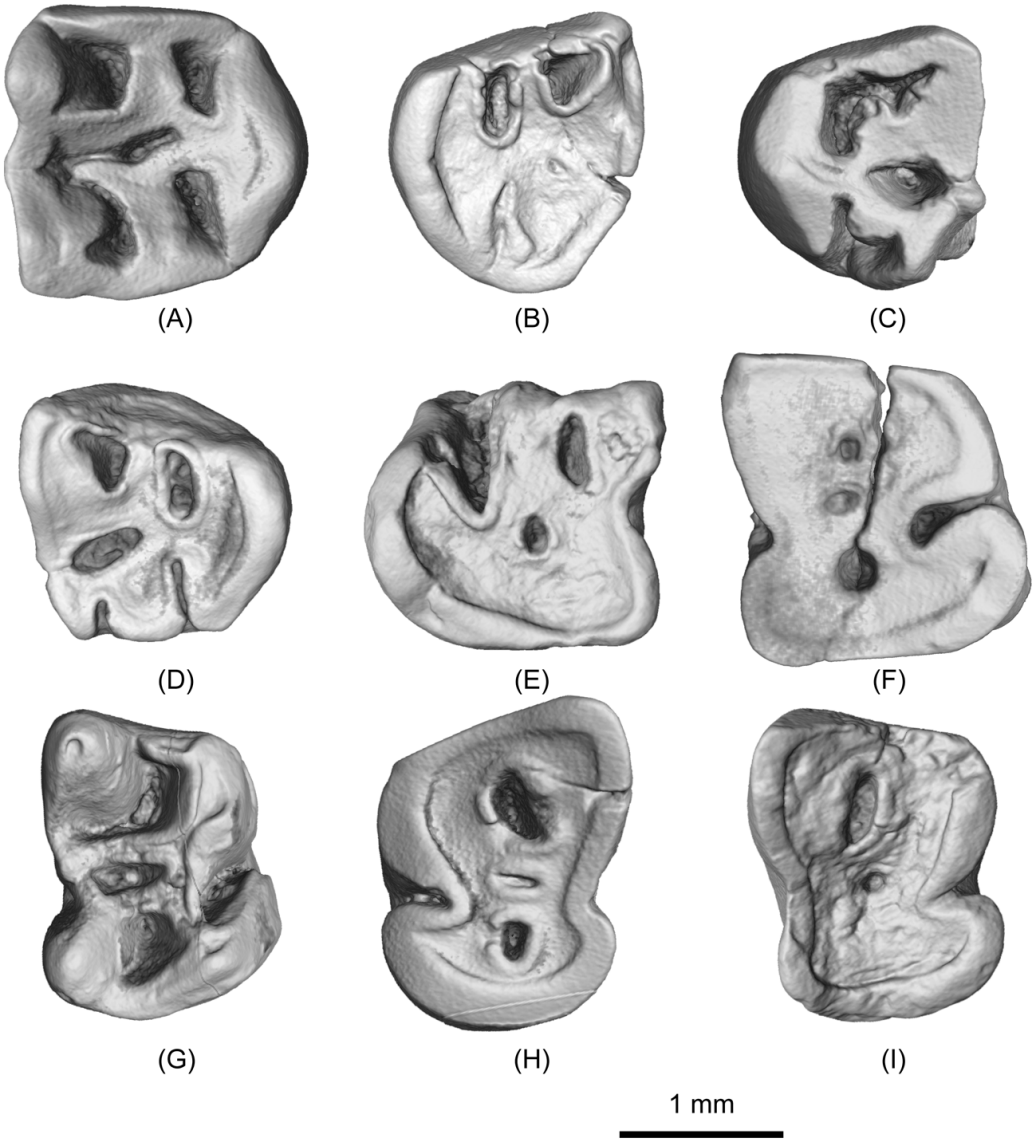
Upper and lower teeth of *Irtyshogaulus major* gen. et sp. nov. (A), V 20809, right M1 (holotype); (B)-(C), V 20810.1–2, left M3s; (D), V 20810.3, right M3; (E), V 20810.4, left p4 (broken); (F), V 20810.5, right m1; (G), V 20810.6, right m2; (H), V 20810.7, left m3; (I), V 20810.8, right m3; Scale bar, 1 mm.

urn:lsid:zoobank.org:act:2FA88DD0-A064-4195-9B23-096DADD09158

(Fig 3; Table 2; S7–S12 Movies)

**Table 2:** Measurements for *Irtyshogaulus major* gen. et sp. nov.

Specimen number	Tooth	Length (mm)	Width (mm)
IVPP V 20809	right M1	1.80	1.78
IVPP V 20810.1	left M3	1.76	1.55
IVPP V 20810.2	left M3	1.53	1.50
IVPP V 20810.3	right M3	1.58	1.58
IVPP V 20810.4	left p4	-	1.84
IVPP V 20810.5	right m1	1.90	1.78
IVPP V 20810.6	right m2	1.82	1.30
IVPP V 20810.7	left m3	1.85	1.49
IVPP V 20810.8	right m3	1.85	1.42

#### Type Specimen

IVPP V 20809, a right M1.

#### Referred Specimens

IVPP specimens: V 20810.1–2, 2 left M3s; V 20810.3, a right M3; V 20810.4, a left p4 (broken); V 20810.5, a right m1; V 20810.6, a right m2; V 20810.7, a left m3; V 20810.8, a right m3.

#### Locality and age

Locality XJ200604, northwestern Junggar Basin, Xinjiang, China. Early Miocene.

#### Diagnosis

Larger than *I*. *minor*. Compared to *I*. *minor*, M1 with thicker crests and narrower fossettes. M3 with well developed paraconule and metaconule. Lower molars with parallel metalophid II and hypolophid, narrower buccal inflection, more significant lingual inflection, and a small mesostylid. Upper and lower molar mesial side with deeper dentin tract.

#### Etymology

The specific epithet refers to the larger size of this species.

#### Description

Only one specimen (the holotype, IVPP V 20809) is identified as an M1 based on the fact that its occlusal surface is symmetrical, and the metaconule is not distally displaced. The tooth is much larger than the M1s referred to *I*. *minor* (Tables 1 and 2). The protocone is buccolingually compressed, with its mesial and distal crests aligned in a straight line. This wall-like protocone is similar to that in *Promylagaulus* and *Trilaccogaulus*, but different from that in *I*. *minor* and *Lamugaulus*. The paracone and metacone are proportionally stouter than those of *I*. *minor*, and more pillar-like. The mesial and distal crests of the paracone and metacone are high and thick, and form a complete wall along the buccal side of the tooth. Between the paracone and metacone is a pillar-like mesostyle. As in other promylagaulines, the mesostyle does not form a sharp ridge. The mesial and distal cingula are straight, high, and relatively thicker than in *I*. *minor*. Two shallow sulci are present at the joints between the mesial cingulum and the protocone, and between the mesial cingulum and the paracone. They suggest the presence of two deep grooves when the tooth was less worn. The paraconule and metaconule are very large (in particular the metaconule) and nearly as large as the metacone. The two cusps are firmly fused with the mesial and distal cingula respectively. Compared with *I*. *minor*, the paraconule and metaconule in *I*. *major* occupy more tooth crown space. The protoloph and metaloph are straight and thick. Proportionally they are thicker than those in *I*. *minor*, and much thicker than in other promylagaulines. Similar to *I*. *minor* and *Lamugaulus*, but different from *Promylagaulus* and *Trilaccogaulus*, the lingual parts of the protoloph and metaloph merge into one crest that originates from the middle part of the buccal side of the protocone. The buccal part of the protoloph connects to the distal crest of the paracone. The buccal part of the metaloph is free. The fossettes of the tooth are all relatively narrower than in *I*. *minor*. The mesiobuccal fossette is the largest and roughly square in outline. The mesiolingual and distolingual fossettes are about the same size, both buccolingually narrow. The distobuccal fossette is a curved fossa, and its buccal side joins the trough-like central fossette.

Three teeth are identified as M3s, and their distal ends are significantly reduced. The distal crest of the protocone is much shorter than the mesial crest. The metacone is very small, and much smaller than the paracone. The mesial cingulum is well developed, but the distal cingulum is absent, leaving the tooth open distally. In *I*. *minor*, the distal cingulum is well developed and completely encloses the distal tooth border. The paraconule and metaconule are present. When it is deeply worn (IVPP V 20810.1), the metaconule becomes a large round cusp, and is much larger than the paraconule and metacone. In a sharp contrast, the metaconule of M3 in *I*. *minor* is reduced and crest-like. Different from *I*. *minor*, in which the protoloph and metaloph merge into one ridge, the M3 protoloph and metaloph in *I*. *major* are present. The protoloph connects the protocone, paraconule, and paracone. The metaloph connects the protocone, metaconule, and metacone. Two mesial fossettes and the central fossette are well defined. The two distal fossettes are not closed distally and as a result are notched.

One broken and deeply worn tooth (IVPP V 20810.4) is identified as p4. The preserved part shows the mesial portion of the tooth is narrower than the distal portion. As in *Lamugaulus*, the p4 of *I*. *major* is not much larger than the m1. In *Promylagaulus* and *Trilaccogaulus*, the p4 is more than two-times larger than the m1. The protoconid is not preserved, and the metaconid bears a very long distal crest. The distal crest of p4 metaconid in *Lamugaulus* is relatively weak, and in *I*. *minor*, *Promylagaulus*, and *Trilaccogaulus* this crest is long and high. The distal portion of the tooth is buccolingually expanded. The hypoconid is very large. Its mesiobuccal side extends into a short blunt ridge, and its distolingual side is continuous with the high and thick distolophid. In other promylagaulines, the mesiobuccal side of p4 hypoconid is quite round. The entoconid is deeply worn and fused with the distolophid. Its mesiobuccal side extends and becomes the hypolophid, running obliquely to join the ectolophid. The buccal inflection is very deep, but its distal end is not expanded. The depth of the buccal inflection likely is correlated with the depth of tooth wear. In *Promylagaulus*, early wear specimens show very deep buccal inflections, and deeply worn specimens exhibit shallow buccal inflections. The lingual inflection is quite obvious. The preserved lingual and distal fossettids are all quite deep. The lingual fossettid is particularly large, nearly double the size of the distal fossettid. Proportionally, the p4 distal fossettid in *I*. *major* is smaller than that in *Promylagaulus* and *Trilaccogaulus*.

A deeply worn tooth (IVPP V 20810.5) is identified as m1 based on its mesiodistally very compressed root. The tooth is close to the size of the p4, and larger than all the other lower molars. Its mesial side is slightly narrower than its distal side. Both borders show straight contact facets. The protoconid, metaconid, hypoconid, and entoconid should all have been quite massive when not worn. Three fossettids gradually increase in size from mesial to distal. The lingual fossettid of m1 is small, differing from the p4. The buccal inflection is a narrow, deep, and curved valley, but the lingual inflection is present as a shallow concavity. No trace of ectomesolophid is present in this valley. In *Lamugaulus*, the tooth identified as m1/2 has a much broader buccal inflection, and in *Trilaccogaulus*, the ectomesolophid plugs in the buccal inflection and partly divides the inflection.

The m2 (IVPP V 20810.6) is roughly rectangular in occlusal view, with its buccal side slightly shorter than the lingual side. As in *I*. *minor*, the protoconid is a blunt crescentic cusp. Its lingual side is strongly concave. The mesial arm of the protoconid extends lingually for a short distance to form the metalophid I. The base of this lophid is fused with the buccal side of the metaconid, but a shallow sulcus between them is still present in the freshly worn specimen as the one (IVPP V 20810.6) described here. The distal arm of the protoconid extends to the distal side of the metaconid and forms the long and thick metalophid II. The metaconid is a conical and sharp cusp. Compared with *I*. *minor*, the base of the m2 metaconid in *I*. *major* is more expanded. Its mesial side is round and smooth, and its distal side tapers into the high metastylid crest. A small but obvious mesostylid is developed at the joint between the metastylid crest and metalophid II. The hypoconid is an obliquely compressed cusp. As in *I*. *minor*, mesiobuccal side of the hypoconid becomes a short blunt crest, and the distolingual side extends as the long and thick distolophid. From the mesiolingual side of the hypoconid, the wall-like ectolophid extends mesially to join the protoconid. Compared to *I*. *minor* and *Lamugaulus*, the m2 ectolophid in *I*. *major* is more buccally positioned. From the middle of the lingual side of the ectolophid, the thick hypolophid runs lingually, parallel to the metalophid II, and joins the mesial side of the entoconid. In *I*. *minor*, the m2 hypolophid is diagonal and smoothly merges into the entoconid instead of being parallel to the metalophid II. The entoconid is as high and sharp as the metaconid, but slightly smaller. The mesial side of the entoconid bears a high ridge that joins the mesostylid and encloses the lingual fossette. In *I*. *minor*, this ridge is short and low, and leaves a shallow notch between the entoconid and the metastylid. The cusps and ridges of the tooth enclose three fossae. The mesial fossettid is a large and curved one, the narrow lingual fossettid is the smallest, and the distal fossettid is round and deep. Given the basal expansion of the metaconid, the m2 mesial fossettid in *I*. *major* is narrower than that in *I*. *minor*. The lingual fossettid is proportionally much narrower than those in *I*. *minor* and *Lamugaulus*. The buccal inflection is narrow and trough-like. The lingual end of the buccal inflection is unexpanded. As in *I*. *minor* and *Lamugaulus*, no ectomesolophid is developed in the buccal inflection. The lingual inflection is a small concavity between the mesostylid and entoconid.

Two deeply worn lower molars (IVPP V 20810.7–8) were identified as m3s. The mesial side of the tooth has a flat interdental wear facet. The distal end is rounded and relatively narrow. In early wear, m3 should have well-developed protoconid, metaconid, hypoconid, and entoconid, as in the other lower molars. The pattern of the ridges should also be similar to m1-2, particularly the metalophid II and hypolophid, which are parallel to each other. Three fossettids (the mesial, the lingual, and the distal) are all present, with the mesial fossettid being the largest, and the lingual fossettid the smallest. The lingual fossettid is mesiodistally narrow, closely resembling the lingual fossettid morphology in m1-2.

### Comparison

*Promylagaulus* has a proportionally much bigger p4 (and probably the corresponding P4 also) than *Irtyshogaulus*. The enamel of the buccal and the lingual side of the molars is similar in thickness in *Promylagaulus*. In *Irtyshogaulus*, in contrast, the enamel of the buccal side is much thicker than that of the lingual side. The upper molars of *Promylagaulus* have a broad distal lingual fossette fused with the central fossette. The lingual part of the metaloph is absent. The upper molars of *Irtyshogaulus* have a more complicated fossette pattern. The mesial, the central, the distal lingual, and the distal buccal fossettes are all well separated. The p4 of *Promylagaulus* has a well developed mesostylid, mesoconid, and hypoconulid, and those cusps are all absent in *Irtyshogaulus*. In the lower molars of *Promylagaulus*, the metalophid II is curved and joins the metaconid instead of the metastylid crest. The metasylid crest of *Promylagaulus* distally extends and is connected to the large mesostylid. A high crest developed from the mesostylid (the mesostylid crest) extends mesiobuccally to join the metalophid II. A deep lingual mesial fossettid is enclosed by the mesostylid crest, the metastylid crest, and metalophid II. In *Irtyshogaulus*, the mesostylid is very small. No mesostylid crest is present, and the mesial fossette is not divided. A mesial crest of the entoconid of *Promylagaulus* is oriented mesiobuccally. The lingual fossettid is separated from the deep lingual inflection by this crest. In *Irtyshogaulus*, the mesial crest of the entoconid is more lingually oriented, resulting in a much shallower lingual inflection.

*Trilaccogaulus* also has proportionally very large P4 and p4, and the p4 of *Trilaccogaulus* has very high metastylid crest. When the tooth is deeply worn, the crest will fuse with the entoconid and leave no lingual inflection. In *Irtyshogaulus*, the metastylid crest of the p4 is relatively weak, and the lingual inflection of p4 is persistent even in very late stages of wear. The lower molars of *Trilaccogaulus* have relatively large mesostylid and mesostylid crests, enclosing a deep mesial lingual fossettid together with the metastylid crest and metalophid II, similar to that in *Promylagaulus*. The ectolophid of the lower molars of *Trilaccogaulus* bears a mesoconid and a mesoconid crest. The crest divides the buccal inflection and encloses a distal buccal fossettid. The lingual inflection tends to be confluent with the lingual fossettid. The distal fossettid is usually the largest. *Irtyshogaulus* does not have the mesostylid, mesoconid, and their associated crests. Therefore, the fossa pattern of the lower molars of *Irtyshogaulus* is simpler than that of *Trilaccogaulus*. The upper molars of *Trilaccogaulus* have relatively slender paraconules and metaconules, and thinner protoloph and metaloph than in *Irtyshogaulus*. As in *Promylagaulus*, the lingual part of the metaloph of *Trilaccogaulus* does not join the protocone, and the central fossette is fused with the distal lingual fossette. The central and distal lingual fossettes of *Irtyshogaulus* are all relatively small.

When compared with the other Asian mylagaulids, *Irtyshogaulus* is much smaller than the early middle Miocene *Tschalimys* and *Simpligaulus*. It is quite close to the early Miocene *Lamugaulus* in size. The upper molars of *Irtyshogaulus* have a squarer occlusal surface than those of *Lamugaulus*. Its upper molar protocones are mesiodistally long. In *Lamugaulus*, the protocone is relatively short and more bulky lingually. The paracone and metacone of *Irtyshogaulus* have thick and high mesial and distal ridges, and the two cusps of *Lamugaulus* are more conical and a deep notch is present between them. The cusps of the lower molars of *Lamugaulus* are also more conical than those of *Irtyshogaulus*. Proportionally, the mesial fossettid of *Lamugaulus* is narrower and more curved than that of *Irtyshogaulus*, the buccal and lingual inflections of *Lamugaulus* are more extensive, and the mesostylid is bigger.

*Crucimys* was referred to the subfamily Promylagaulinae [7], but the phylogenetic analysis of Hopkins [8] positioned the taxon in the paraphyletic Meniscomyinae. The p4 and m1 of the only species *Crucimys milleri* have a relatively weaker metastylid crest that does not join the mesostylid distally. Therefore, the mesial fossettid on the p4 and m1 in *Crucimys* is not fully closed on the lingual side. The mesostylid in *Crucimys* is a distinct pillar-like cusp. It is more distally positioned relative to the protoconid, and the metalophid II is more distally directed. The entoconid in *Crucimys* is relatively small. It is confluent with the hypolophid mesiobuccally. The lingual fossettid between the metalophid II and hypolophid is closed lingually only in the deeply worn teeth. The distal fossettid in *Irtyshogaulus* is a rounded deep fossa. In *Crucimys*, this fossa is mesiodistally narrow. The mesoconid, a small cusp developed on the ectolophid, is well developed in *Crucimys*. A short spur develops from this cusp, (the mesoconid crest), which extends buccally to the mesial extension of the hypoconid. A small distal buccal fossettid is enclosed between the mesoconid crest and the hypoconid. A mesoconid, mesoconid crest, and distal buccal fossettid are present in many promylagaulines, but absent in *Irtyshogaulus*.

*Brachygaulus* was suggested to be close to the ancestral form of Mylagaulidae [15]. Three named species and one unnamed species of *Brachygaulus* all have a much lower crown than the more derived mylagaulids. In *Brachygaulus*, the size of P4/p4 is similar to the upper/lower molars. Mylagaulids all have bigger P4s and p4s relative to their molars. Only an incomplete p4 is known for *Irtyshogaulus major*. The broad distal part of this tooth shows that it is bigger than the lower molars. In *Brachygaulus*, the paracone and metacone of the upper molars are mesiodistally compressed, and the paraconule and metaconule are conical. In *Irtyshogaulus*, the paracone, metacone, paraconule, and metaconule are all buccolingually compressed. In *Brachygaulus*, the valley between mesial cingulum and protoloph, and between protoloph and metaloph are not closed buccally. Therefore, the mesial and central fossettes are not formed in *Brachygaulus*. In *Irtyshogaulus*, these two fossettes are fully closed by the mesial and distal extension of paracone and metacone. In *Brachygaulus*, the p4 and lower molars have low metastylid crests. The mesotylid is absent or present as the lingual terminus of the metalophid II. The connection between metastylid crest and mesostylid/metalophid II is very low or absent. In *Irtyshogaulus*, the metastylid crest is high and thick and a conical mesostylid is present. In *Brachygaulus*, the valleys, between metalophid I and II, between metalophid II and hypolophid, and between hypolophid and distolophid are mesiodistally narrow and buccolingually wide. Their lingual sides are not closed. In *Irtyshogaulus*, these three valleys are fully closed on the lingual tooth side, and mesiodistally expand into deep pits. The mesoconid is big in *Brachygaulus*, but is absent in *Irtyshogaulus*.

### Phylogeny

**Fig 4:**
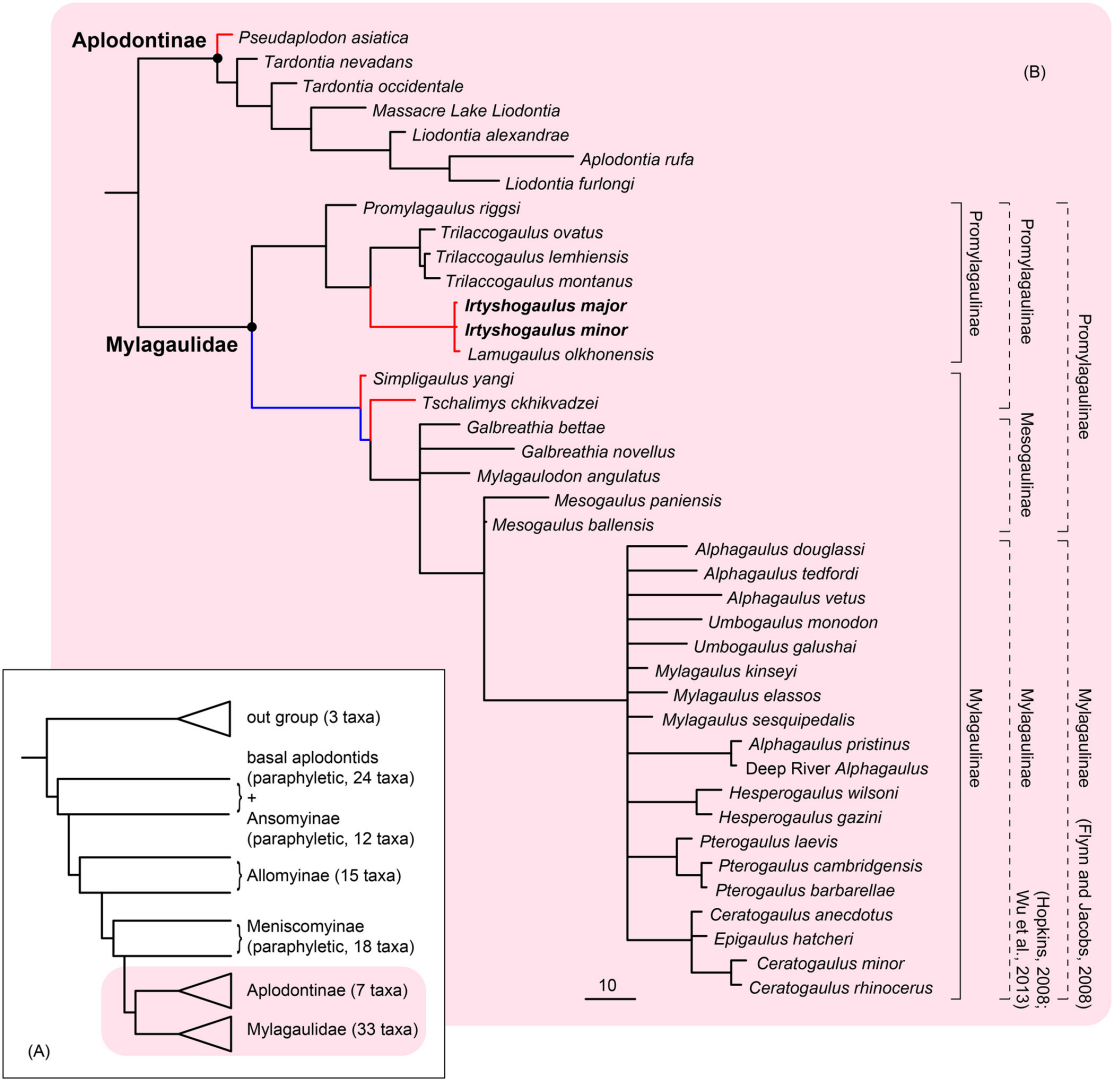
Phylogeny and distribution of mylagaulid and aplodontine rodents. Strict consensus is from 58 most-parsimonious trees. Each tree has a Best Score of 1585, a Consistency Index of 0.1684 and a Retention Index of 0.6659. (A), Summarized phylogeny of Aplodontoidea, showing the phylogenetic positions of Mylagaulidae and Aplodontinae; (B), Phylogeny of Mylagaulidae and Aplodontinae. Branch lengths indicate the number of character changes, but have no temporal meaning. The paleobiogeographic distribution of mylagaulids and aplodontines was reconstructed by using parsimony criterion in Mesquite v3.03: black for North America, red for Asia, and blue for equal parsimony. Dashed square brackets on the right show the previously published classifications, and solid line brackets show the classification scheme used in this paper.

Our phylogenetic analysis based on the updated data matrix (S1 Text) of Hopkins [8] generated 58 most parsimonious trees. The strict consensus of these trees indicates that *Irtyshogaulus* and *Lamugaulus* form a clade between *Promylagaulus* and *Trilaccogaulus* (Fig 4). The four genera comprise a monophyletic group. The Asian genus *Tschalimys* was grouped in the paraphyletic Aplodontidae in the analysis of Hopkins [8]. Here, we updated the coding of *Tschalimys* based on newly discovered specimens (IVPP V 17928, 8107) from the early Middle Miocene Halamagai Formation in northern Junggar Basin of Xinjiang. We found *Tschalimys* and *Simpligaulus* are the most basal taxa of a monophyletic group that includes taxa traditionally referred to subfamilies Mesogaulinae and Mylagaulinae. The taxa of Mylagaulinae consist of a well-defined monophyletic group, but those of Mesogaulinae are paraphyletic.

The monophyly of the Mylagaulidae is well supported in our analysis, but Aplodontidae becomes a paraphyletic group. Within the Family Aplodontidae, only the monophyly of Subfamily Aplodontinae is supported. The Subfamily Aplodontinae is the sister of the monophyletic Mylagaulidae. The two groups were referred to a node-based group Homalodontia Hopkins, 2008.

## Discussion

Mylagaulidae is one of the two families classified in the superfamily Aplodontoidea [6,7,15,20]. Our phylogenetic analysis based on new discoveries and updated scorings supports the monophyly of Mylagaulidae, but reveals the paraphyletic status of Aplodontidae. A thorough taxonomic revision of the family Aplodontidae is needed, but it is beyond the scope of this paper.

The Family Mylagaulidae has been divided into three subfamilies [4,8] including Promylagaulinae Rensberger, 1980, Mesogaulinae Korth, 2000, and Mylagaulinae Cope, 1881, or only into two subfamilies [7] including Promylagaulinae Rensberger, 1980 and Mylagaulinae Cope, 1881. In the classification of Hopkins [8], Promylagaulinae is a paraphyletic group and includes only *Promylagaulus* and *Trilaccogaulus*. In the classification of Flynn and Jacobs [7], *Mesogaulus*, *Galbreathia* and *Mylagaulodon*, were also assigned to the Promylagaulinae, but Hopkins [8] and Korth [4] placed those genera in the Mesogaulinae. Previously the Asian mylagaulids *Simpligaulus*, *Tschalimys* and *Lamugaulus* usually were attributed to the Subfamily Promylagaulinae [7,10,11]. Hopkins [8] excluded *Tschalimys* from Mylagaulidae. Our analysis suggests that *Lamugaulus* and *Irtyshogaulus* are closely related taxa. Both fall in the monophyletic group including *Promylagaulus* and *Trilaccogaulus*. *Tschalimys*, *Simpligaulus*, and the taxa referred to Mesogaulinae and Mylagaulinae form a monophyletic group. Our analysis also demonstrates that both Promylagaulinae sensu Flynn and Jacobs [7] and Mesogaulinae sensu Hopkins [8] and Korth [4] are paraphyletic. Based on these results, we suggest to refer *Lamugaulus*, *Irtyshogaulus*, *Promylagaulus* and *Trilaccogaulus* to the revised Subfamily Promylagaulinae, and refer the other mylagaulids to the Subfamily Mylagaulinae (Fig 4).

The origin of mylagaulids is unclear. Korth and Tabrum [15] suggested that mylagaulids may have originated from a North American prosciurine-like ancestor such as *Brachygaulus*. However, our phylogenetic analysis reveals that *Brachygaulus* is a basal taxon of the paraphyletic Meniscomyinae, distantly related to the Mylagaulidae clade (S2 Text). We therefore have no reason to believe that *Brachygaulus* is similar to the common ancestor of mylagaulids. Instead, the phylogenetic analysis of Hopkins [8] and our analysis based on an updated data matrix both demonstrate a sister-relationship between Aplodontinae and Mylagaulidae. The last common ancestor of the Aplodontinae-Mylagaulidae clade should present characters shared by both early Aplodontinae and Mylagaulidae (Fig 4). Any taxon of Aplodontinae or Mylagaulidae clade that conserves the most plesiomorphic characters should be most similar to the ancestral morphotype of the two clades.

When the geographic occurrences of the ancestors of Mylagaulidae, Promylagaulinae and Mylagaulinae are reconstructed with the parsimony criterion (Fig 4), the results indicate North American origins of Mylagaulidae and Promylagaulinae, but the geographic origin of Mylagaulinae is uncertain. Asian promylagaulines (*Irtyshogaulus* and *Lamugaulus*) share a common ancestor that came from North America. Both Asian and North American origins of Mylagaulinae are equally parsimonious. *Mylagaulodon angulatus* is the oldest-known mylagauline rodent and was discovered from the early Miocene in North America. The Asian mylagaulines, *Tschalimys* and *Simpligaulus*, were discovered from the early Middle Miocene Sarybulak Formation in Kazakhstan and the Halamagai Formation in China, roughly 17–14 Ma. The Asian origin hypothesis of mylagaulines would imply a long (at least 6 million years) ghost lineage for Asian mylagaulines. In northern and central Asia, particularly in Junggar Basin and Inner Mongolia in China, the early and middle Miocene mammalian fossil sites have been intensively quarried and sampled, but no mylagauline older than *Tschalimys* and *Simpligaulus* has ever been discovered. The North American origin hypothesis of mylagaulines would imply that some basal mylagaulines immigrated into Asia in early Middle Miocene, slightly later than or roughly at the same time as the arrival of promylagaulines (*Irtyshogaulus* and *Lamugaulus*). Our phylogenetic analysis indicates that *Tschalimys* and *Simpligaulus* are very close to *Mylagaulodon*, *Galbreathia*, and *Mesogaulus*. If the American origin hypothesis is correct, then the ancestor of Asian mylagaulines should be somewhat similar to *Mylagaulodon*, *Galbreathia*, and *Mesogaulus*. We believe it is quite likely.

## Supporting Information

S1 Movie *Irtyshogaulus minor* left M1 (IVPP V 20328) xy rotation. (MOV)

S2 Movie *Irtyshogaulus minor* left M1 (IVPP V 20328) xz rotation. (MOV)

S3 Movie *Irtyshogaulus minor* left M1 (IVPP V 20328) cut. (MOV)

S4 Movie *Irtyshogaulus minor* left m2 (IVPP V 20329.14) xy rotation. (MOV)

S5 Movie *Irtyshogaulus minor* left m2 (IVPP V 20329.14) xz rotation. (MOV)

S6 Movie *Irtyshogaulus minor* left m2 (IVPP V 20329.14) cut. (MOV)

S7 Movie *Irtyshogaulus major* right M1 (IVPP V 20809) xy rotation. (MOV)

S8 Movie *Irtyshogaulus major* right M1 (IVPP V 20809) xz rotation. (MOV)

S9 Movie *Irtyshogaulus major* right M1 (IVPP V 20809) cut. (MOV)

S10 Movie *Irtyshogaulus major* right m2 (IVPP V 20810.6) xy rotation. (MOV)

S11 Movie *Irtyshogaulus major* right m2 (IVPP V 20810.6) xz rotation. (MOV)

S12 Movie *Irtyshogaulus major* right m2 (IVPP V 20810.6) cut. (MOV)

S1 Text Data matrix used in phylogenetic analysis in TNT format. (TXT)

S2 Text Majority and strict consensus trees in NEXUS format. (TXT)
